# Supplementary material for: The conformational plasticity of the selectivity filter methionines controls the in-cell Cu(I) uptake through the CTR1 transporter
Source: QRB Discov. 2022 Apr 21;3:e3. doi: 10.1017/qrd.2022.2 (PMC10392627; doi:10.1017/qrd.2022.2)
Supplement: Supplementary file 1 [file qrdsup.zip › S2633289222000023sup003.pdf]

Supporting Information

The Conformational Plasticity of the Selectivity Filter Methionines Controls the In-Cell Cu(I) Uptake through the CTR1 transporter

Pavel Janoš,[a] Jana Aupič,[a] Sharon Ruthstein,[b] and Alessandra Magistrato[a*]

[a] CNR-IOM c/o International School for Advanced studies (SISSA/ISAS), via Bonomea 265, 34136, Trieste, Italy

[b] Department of Chemistry, Faculty of Exact Sciences, and the, Institute for Nanotechnology and Advanced Materials (BINA), Bar-Ilan University, 5290002, Ramat-Gan, Israel

Table of Content

Methods:

Model building 3
QM/MM MD simulations 3
QM/MM metadynamics simulations 4
Classical metadynamics simulations 5
Analyses 6

Supporting Figures:

Figure S1. Cu(I)-S distances........ 7
Figure S2. Cu(I)-Cu(I) (Å) distance 8
Figure S3. Structures of the Met triads from experimental structure and QM/MM........ 9
Figure S4. RMSD of the Site 1 and Site 2........ 9
Figure S5. RMSF of the Site 1 and Site 2........ 10
Figure S6. Cu(I) translocation from Site 1 to Site 2; CV, Met angle and distance evolution........ 11
Figure S7. Radical distribution function of the Cu(I)-O_{water} distance........ 12
Figure S8. Cu(I) dissociation from the Site 2 in absence of second Cu(I); CV, Met angle and distance evolution........ 13
Figure S9. Free energy profiles of the Cu(I) dissociation from the last S2-Met in the absence of second Cu(I)........ 14
Figure S10. Cu(I) dissociation from the Site 2 in presence of Site 1 Cu(I); CV, Met angle and distance evolution........ 15
Figure S11. Free energy profiles of the Cu(I) dissociation from the last S2-Met in the presence of Site 1 Cu(I)........ 16
Figure S12. Free energy profile of the Cu(I) dissociation from the Site 2 in presence of Site 1 Cu(I) with all S2-Mets restricted to IP-conformation........ 17
Figure S13. Free energy surfaces of the O- to IP-conformational flipping from classical MTD simulation........ 18

Supporting Movies:

Movie M1. Translocation of Cu(I) from Site 1 to Site 2........ 19
Movie M2. Dissociation of Cu(I) from Site 2........ 19

References........ 20

Methods

Model building

The structure of CTR1 comprising the transmembrane part and part of the C-term (residues 41-186) was taken from the crystallographic structure PDB entry: 6M98 from *Salmo salar* (Figure 1A) (1). The chaperone apocytochrome b_{562}RIL (BRIL), present in the crystal structure, was replaced by the native loop modelled with the MODELLER program (2). On the basis of this structure we built three models: one with only one Cu(I) ion bound to the most extracellularly exposed Met150 (M154 in hCTR1) triad (Site 1), one with a Cu(I) bound only to the intracellularly exposed Met146 (M150 in hCTR1) triad (Site 2) and one with two Cu(I) ions bound to Site 1 and Site 2 as observed in the crystal structure (Figure 1B).

CHARMM-GUI (3, 4) was used to insert the CTR1 protein into a lipid bilayer and create the simulation box. The orientation of CTR1 with respect to the membrane bilayer was determined using PPM webserver (5). The resulting membrane bilayer consisted of 95 phosphatidylcholine (POPC) molecules in each leaflet. The simulation box dimensions were 90x90x110 Å and further contained 17446 water molecules and 0.15 M concentration of NaCl (45 Na^+ ions, 50 Cl^- ions). The protein was described using Amber FF14SB forcefield (6) while for the POPC bilayer we used lipids17 FF (7). Water was described using the TIP3P model (8). Joung and Chetham parameters (9) were used for Na^+ and Cl^- ions. Cu FF parameters were taken from Merz and coworkers (10) devised for MD simulations of Cys coordinating Cu-proteins. The system was minimized using 1000 steps of steep descent optimization with distance restraints on the S-Cu(I) coordination bonds with value 2.25 Å and force constant 5000 kJ/mol·nm. For the rest of the equilibration procedure the positions of Cu(I) ions and the methionine triads coordinating them were restrained using matrix of distance restraints with a force constant 2000 kJ/mol·nm. Gromacs 2018 was used for the classical MD simulations (11). The system was equilibrated in 8 steps with an increasing MD simulations length (0.5 ns, 0.25 ns, 0.5 ns, 0.5 ns, 0.5 ns, 0.5 ns, 0.5 ns, 2.0 ns) and decreasing restraints on the protein (5000 kJ/mol·nm, 4000 kJ/mol·nm, 3000 kJ/mol·nm, 2000 kJ/mol·nm, 1000 kJ/mol·nm, 500 kJ/mol·nm, 250 kJ/mol·nm, 50 kJ/mol·nm, 0 kJ/mol·nm) and the membrane (2000 kJ/mol·nm, 2000 kJ/mol·nm, 1000 kJ/mol·nm, 500 kJ/mol·nm, 250 kJ/mol·nm, 100 kJ/mol·nm, 50 kJ/mol·nm, 0 kJ/mol·nm, 0 kJ/mol·nm). Restraints on Cα atoms with 50 kJ/mol·nm were applied in the last step. The Cα restraints were kept for the first 30 ns of MD simulations to allow for membrane equilibration. 100 ns-long classical MD simulations of each model were performed before switching to QM/MM MD simulations.

QM/MM MD simulations

The QM/MM MD simulations were performed using CP2K version 6.1 (12). The QM zone comprised all six Met residues (cut at the C -Cβ bond) forming the selectivity filter and one or two

Cu(I) ions along with nearby water molecules. The QM zone of the system with the unoccupied bottom Met146-triad (used to study the Cu(I) translocation from Site 1 to Site 2) included 10 water molecules, which were close to Cu(I). These were periodically updated as the Cu(I) approached Site 2. Conversely, the QM zones of the simulations done to study the dissociation of Cu(I) from Site 2 towards the interval vestibule (comprising either two Cu(I) ions or only one Cu(I) in Site 2) included 8 waters molecules, which were restrained with an upper wall 6 Å distance restraint to the Site 2 Met146-triad defined by a geometric center of its Met C atoms. Additional restraint was placed on nearby MM water molecules with a lower wall distance of 6 Å to the Site 2 Met-triad to prevent the MM waters to exchange with the QM ones. The Plumed 2.7.0 plugin was used for the restraints (13).

The QM zone was described using BLYP functional (14–16) with dual Gaussian-type/plane waves basis set as in previous simulations (17, 18). Specifically, a double-ζ (MOLOPT) basis set (19) was used with an auxiliary PW basis set using the Goedecker–Teter–Hutter (GTH) pseudopotentials (20) and a plane wave cutoff of 320 Ry. DFT-D3 dispersion correction was applied (21). After switching to QM/MM MD the system was relaxed through series of alternating short unbiased NVE and simulated annealing simulations using Langevin thermostat. Next, the system was gradually heated to 300 K over the course of 10 ps and left to equilibrate during a 10 ps unbiased QM/MM MD. The MD timestep was 0.5 fs. The heating and production runs used two canonical sampling through velocity rescaling (CSVR) thermostats (22): one applied to the QM part and the other to the rest of the system. Time constant of both was 100 fs.

QM/MM metadynamics simulations

The metadynamics (MTD) simulations were exploited to study the mechanism of Cu(I) translocation through the CTR1 selectivity filter. To this aim we employed the MTD implementation of CP2K using two collective variables (CVs): CV1 – distance of the Cu(I) to the plane of the Site 1 Met-150 triad, defined on the bases of the Met150 C atoms; CV2 defined as the coordination number (CN) of the Cu(I) with respect to Sulphur (S) atoms of the Site 2 Met146-triad similarly to a previous study.(23) For the exponential form of coordination number we used the p=8, q=16 and d_0=2.85 Å parameters. The width of the deposited MTD hills was 0.5 Å for CV1 and 0.05 for CV2, while the height was 1 kcal/mol and the deposition time was 50 fs. The effect of the MTD settings on the results was assessed by performing replicas MTD simulations with smaller hill height (0.6 kcal/mol) and longer deposition time (100 fs).

The dissociation of the Cu(I) ion from the Site 2 Met146-triad was simulated with the MTD implementation encompassed in Plumed 2.7.0 using again two CVs: CV1 – accounting for the z-projection of the distance of the Cu(I) to the center of the CTR1 selectivity filter defined using C atoms of all Site 1 and Site 2-Mets; and CV2 – defined as coordination number (CN) of the Cu(I)

with respect to S atoms of the Site 2 Met146-triad. In this case the width of the deposited hills was 0.5 Å and 0.05, respectively for CV1 and 2, the height was 1 kcal/mol and the deposition time was 50 fs. The final dissociation step from the partially dissociated state, in which Cu(I) is still coordinated to one Site 2-Met, was explored with a separate set of MTD simulations using only one CV (CV1), accounting for the distance of the Cu(I) ion to the S atom of the last coordinated Site 2-Met. Restraints were put on the coordination number to the Site 2 Met146-triad at 1.15 value and the distance to the other Mets at 3.0 Å with a force constant 1000 kJ/mol·nm. The width of the deposited MTD hills was 0.2 Å, the height was 0.25 kcal/mol and the deposition time was 100 fs. When studying the Cu(I) dissociation from the CTR1 selectivity filter in a CTR1 model containing a single Cu(I) bound to Site 2, two replicas of the final MTD simulation were run to obtain more accurate free energy barrier. Conversely, when studying the same process for a CTR1 model hosting two Cu(I) ions, two replicas of the final dissociation MTD simulation were run, one with restraint on the χ_1 and χ_3 torsional angles of all Site 2-Mets to force them into the in-plane (IP)-conformation, and one unrestrained. This was done to explore the role of the water access to the Site 2 on the free energy barrier of Cu(I) in cell release.

Classical metadynamics simulations

Using the QM/MM MD-equilibrated structure we derived FF-parameters of the CTR1 Met Triads using the Metal Center Protein Builder (mcpb) (24). Both Cu-occupied Met-triads were included in the "small model" used to parametrization the Cu(I) ion coordination sphere. The geometry optimization was done using Gaussian 09 at the B3LYP/6-31G* level with constraints on the distance between the Site 1 and Site 2 Mets. The resulting FF parameters were employed to equilibrate again the system using the previously described MD protocol and to explored the conformational behavior of the selectivity filter Met triads. Specifically, we performed MTD simulation to monitor the torsion angle behavior of the Mets. These can assume three different conformations within the selectivity filter: (i) Outward (O) with the S-Methyl bond parallel to the z-axis of the selectivity filter and the methyl pointing to away from the selectivity filter (i.e. towards the extracellular side for Site 1 and towards intracellular CTR1 vestibule for Site 2), (ii) In-Plane (IP) in which the S-Methyl bond is perpendicular to the z-axis of the filter and the Methyl group is pointing outside from the selectivity filter, and (iii) Inward (I) with the S-Methyl bond is parallel to the z-axis of the selectivity filter and with the methyl pointing inside the selectivity filter (Figure 1C).

In order to calculate the free energy cost for the conformational flipping between the IP and O state of one Site 2-Met the other two Site 2-Mets were blocked in their IP configuration by restraining the χ1 ($_{n-1}$C-Cα-Cβ-Cγ) and χ3 (Cβ-Cγ-S-Cε) torsional angles to the range 2.77 – 3.14 rad. The conformational behavior of the third Site 2-Methionine was instead monitored by biasing

$\chi1$, $\chi2$ (Cα-Cβ-Cγ-S) and $\chi3$ torsion angles in a well-tempered MTD simulation using $\sigma=\pi/18$, height = 0.25 kcal/mol, bias factor 10 and deposition time 5 ps.

Analyses

For analyses purposes the three possible conformational states of Mets were defined, using the angle θ between two vectors: the first is the vector lying between geometric centers of Site 1 and Site 2 Met backbone atoms (Figure 1C), which represents the axis of the selectivity filter, the second vector is defined by Cγ-Cε Met atoms (Figure 1C). The Site 1 conformations are defined with θ angle ranges: 0-40° for the Outward (O), 50-90° for In-Plane (IP) and 100-160° for Inward (I) conformations of Met154 residues. The Site 2 conformations are defined with θ angle ranges: 100-160° for the Outward (O), 50-90° for In-Plane (IP) and 0-40° for Inward (I) conformations of the Met150 residues. The θ angle analysis along with that of the RMSD, RMSF, and radial distribution function was performed using cpptraj (25). The histograms of the θ angle distribution were created by numpy and density normalized (26). Met1-3 labels are used to refer to Mets coming from monomeric chain 1-3. In all MTD simulations the error of the free energy profiles was calculated as the standard deviation of different time averages from different simulation blocks. The minimum free energy paths were obtained using Metadynminer (27).

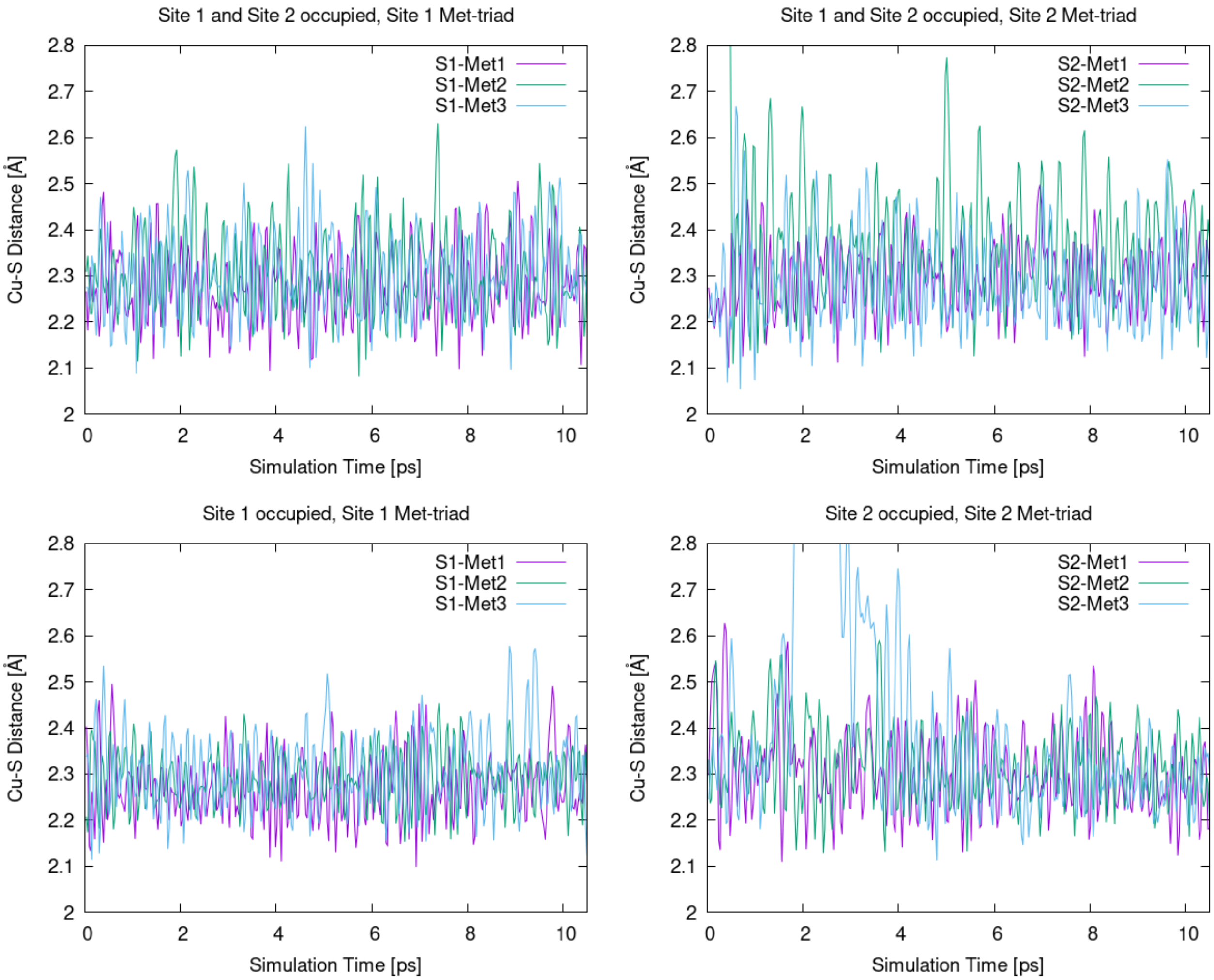

Figure S1. Cu(I)-S distances (Å) vs QM/MM molecular dynamics simulation time (ps) from the methionine residues of the CTR1 selectivity in Site 1 (top or most extracellular oriented) and Site 2 (bottom, most cytosol oriented) triad with both triad binding a Cu(I) ion (A and B), with a Cu(I) bound only to Site 2 (C) and only to Site 1 (D).

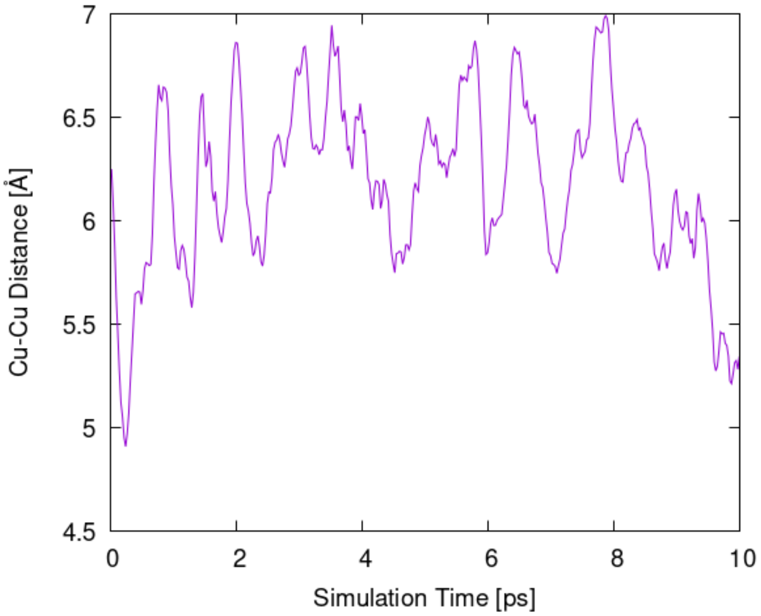

Figure S2. Cu(I)-Cu(I) (Å) distance vs QM/MM molecular dynamics simulation time (ps) in the CTR1 model with both Met-triads occupied by Cu(I) ions.

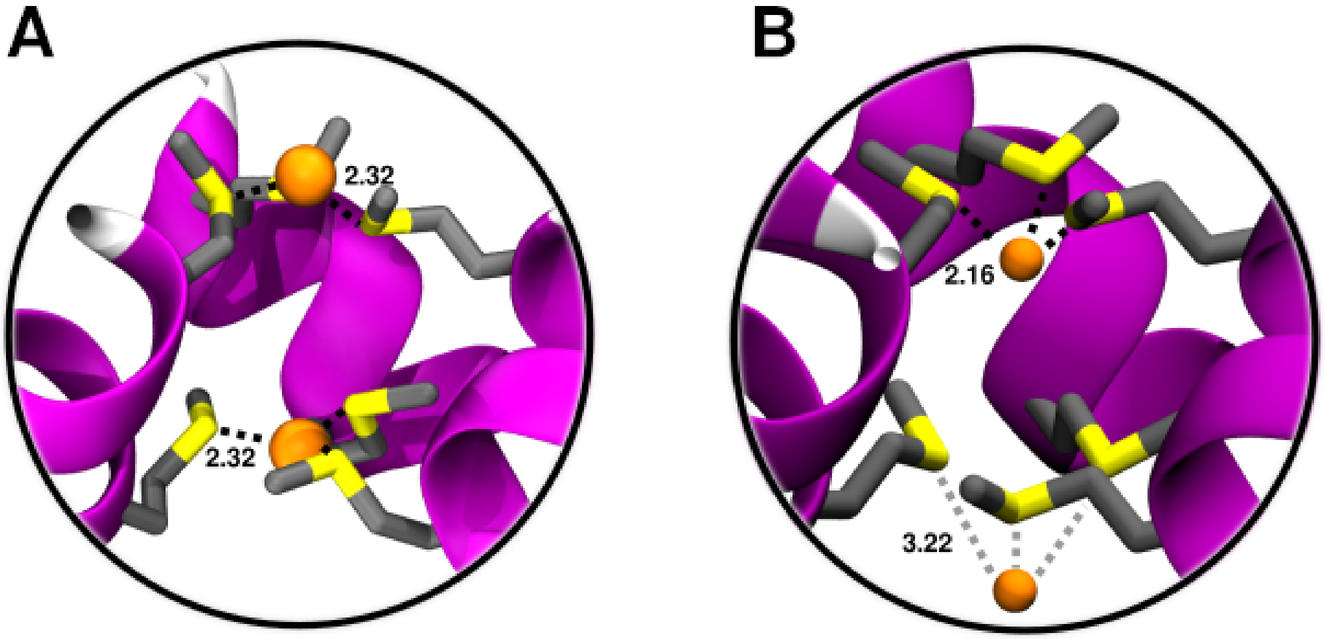

Figure S3. A) Structure of the Site 1 (upper) and Site 2 (lower) Met-triads each coordinating a Cu(I) ion as obtained from QM/MM molecular dynamics simulation. B) Structure of the Met-triads each binding a Cu(I) as obtained from the X-ray structure (PDB ID: 6m98).

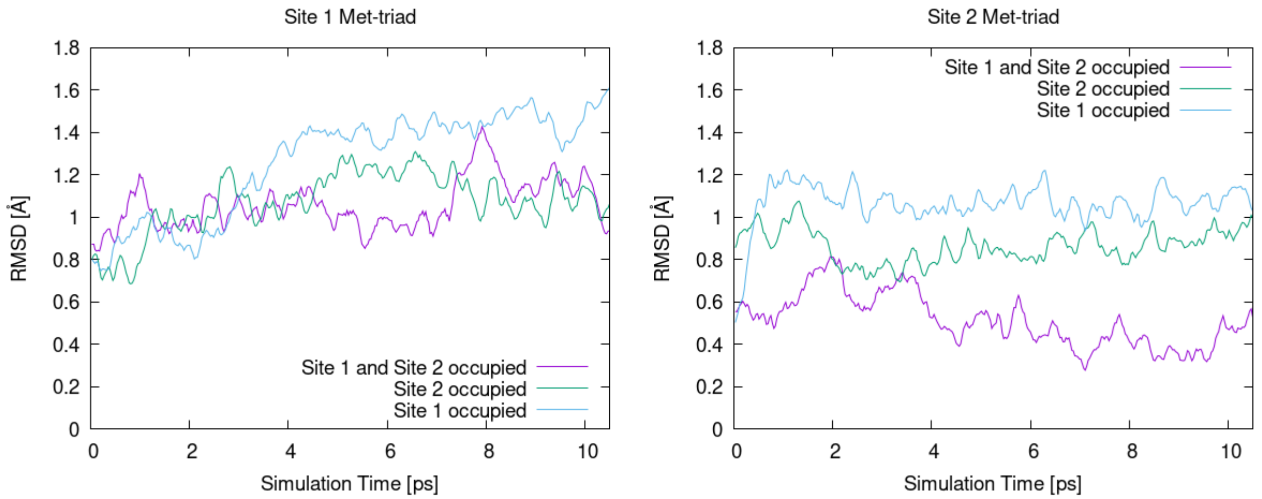

Figure S4. Root Mean Square Deviation (Å) RMSD vs simulation time (ps) of Site 1- (A) and Site 2- (B) Met-triads with respect to the initial conformation as obtained from QM/MM MD simulations. The

CTR1 model with two Cu(I) ions, with Cu(I) bound to Site1 and with Cu(I) bound to Site 2 are shown in magenta, light blue and green lines, respectively.

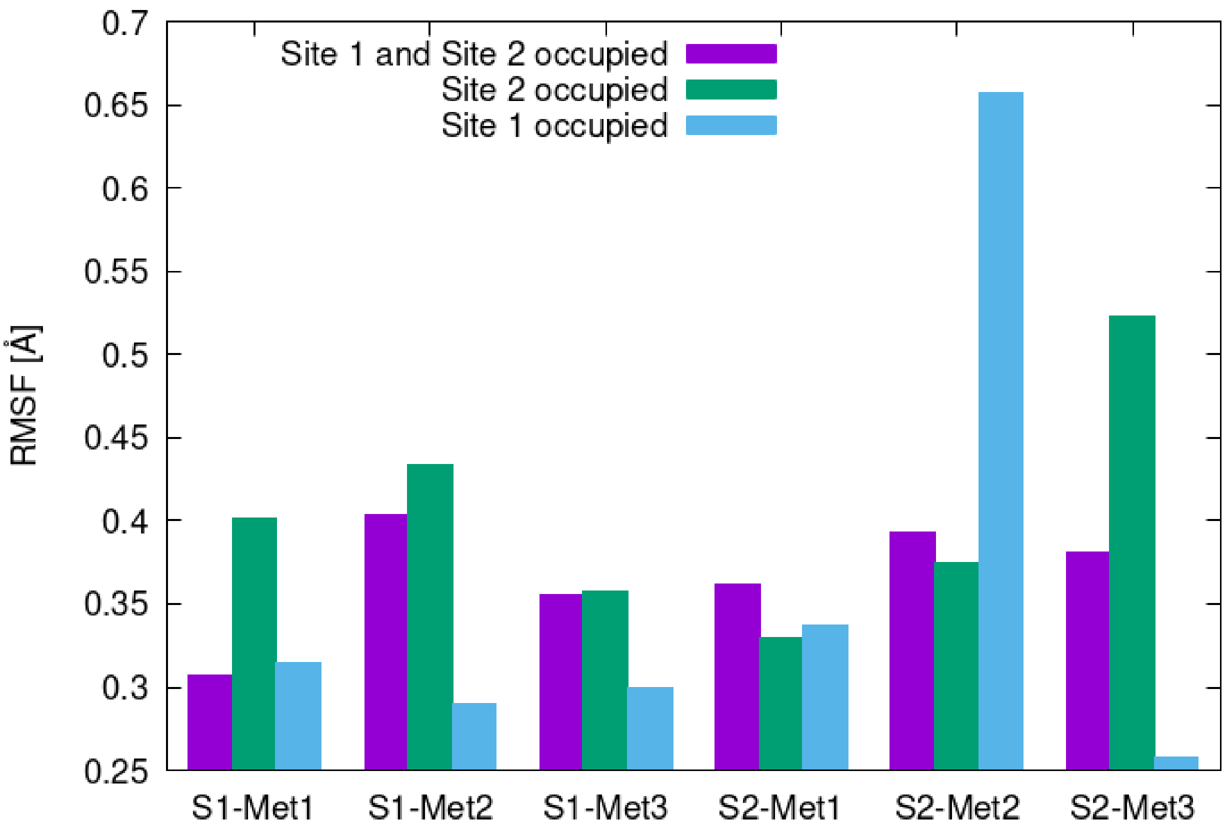

Figure S5. Root Mean Square Fluctuation (RMSF, Å) of the Met residues forming the Site 1 (S1-Met1-3) and Site 2 (S2-Met1-3) Met-triads. The CTR1 model with two Cu(I) ions, with Cu(I) bound to Site1 and with Cu(I) bound to Site 2 are shown in magenta, light blue and green lines, respectively.

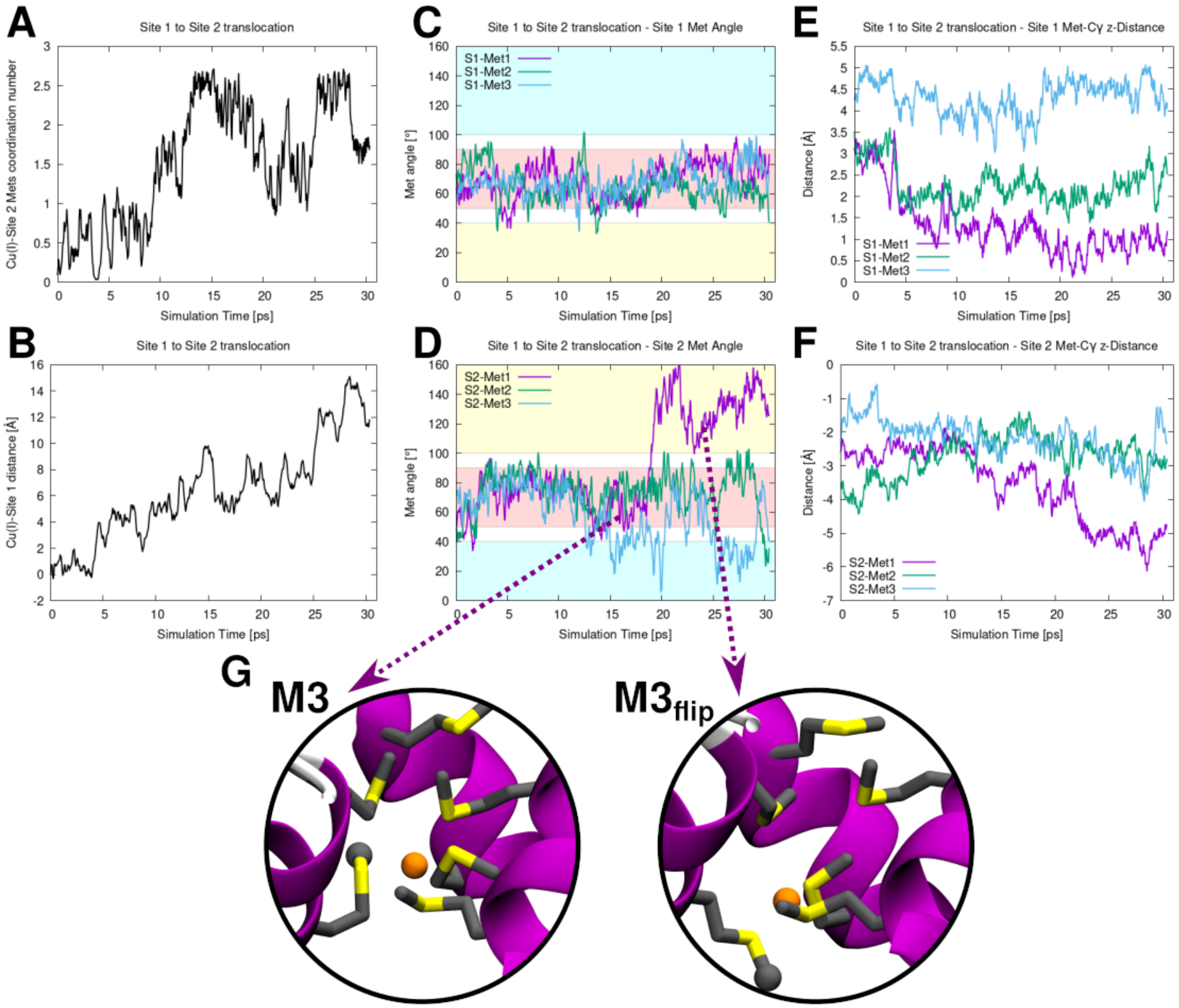

Figure S6. Cu(I) translocation from Site 1 to Site 2. Evolution of the Collective variables (CVs) used in the Metadynamics simulations vs simulation time (ps): (A) Coordination number of the C(I) ion to the S atoms of the Site 2 Met triad; (B) Distance of the Cu(I) to the S-Met atoms of the Site 1-Met triad; Conformations of the Site 1 (C) and Site 2 (D) Mets measured as the θ angle (°) between the Met Cγ-Cε vector and the vector of the selectivity filtered defined by geometric centers of Site 1 and Site 2 backbone atoms (see Figure 1C of the main text). S1-Met1-3 and S2-Met1-3 refer to the methionines of the top (extracellular matrix-exposed Met-triad) and bottom (intracellular-exposed Met-triad), respectively. Areas of the plots corresponding to the inward, in-plane and outward conformations are highlighted in cyan, red and yellow, respectively. Z-projection of distance (Å) of the Site 1 (E) and Site 2 (F) Met@Cγ to the center of the selectivity filter defined as the center of Site 1 and Site 2

Met@Cα atoms. (G) Close-ups of the states corresponding to the conformational change of the Site 2-Met1.

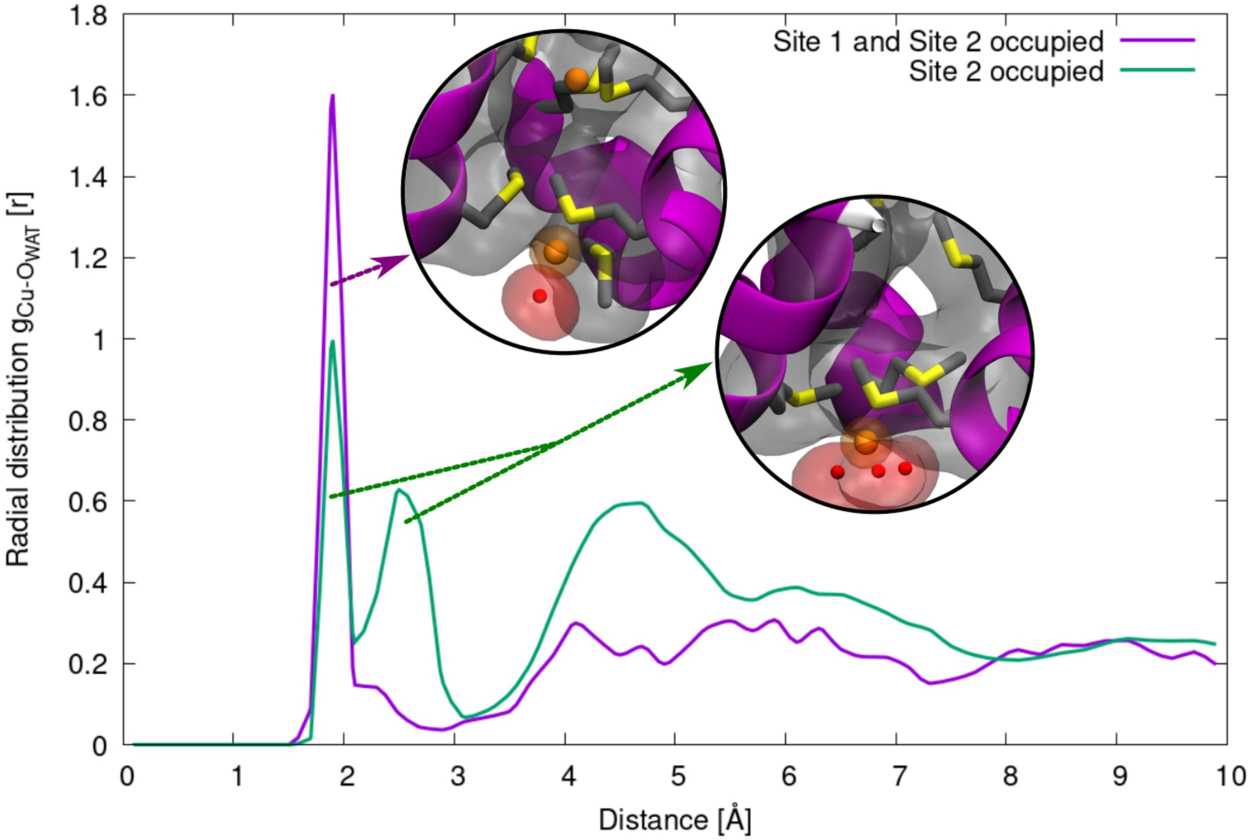

Figure S7. Radial distribution function (RDF) of the Cu(I) ions (bound to Site 2) – O_{WAT} (oxygen atom of water molecules) distance in the model where both Site 1 and Site 2 bind a metal ion (purple line) and when only Site 2 binds a Cu(I) ion (green line). Inlaid pictures show close-up of the corresponding CTR1 conformations. CTR1 is shown as magenta new cartoons, the Met-triads are depicted as licorice and Cu(I) and water oxygen atoms as orange and red spheres, respectively. Hydrogens are omitted for clarity. CTR1 is highlighted as gray surface while water molecules and Cu(I) as red and orange surface, respectively.

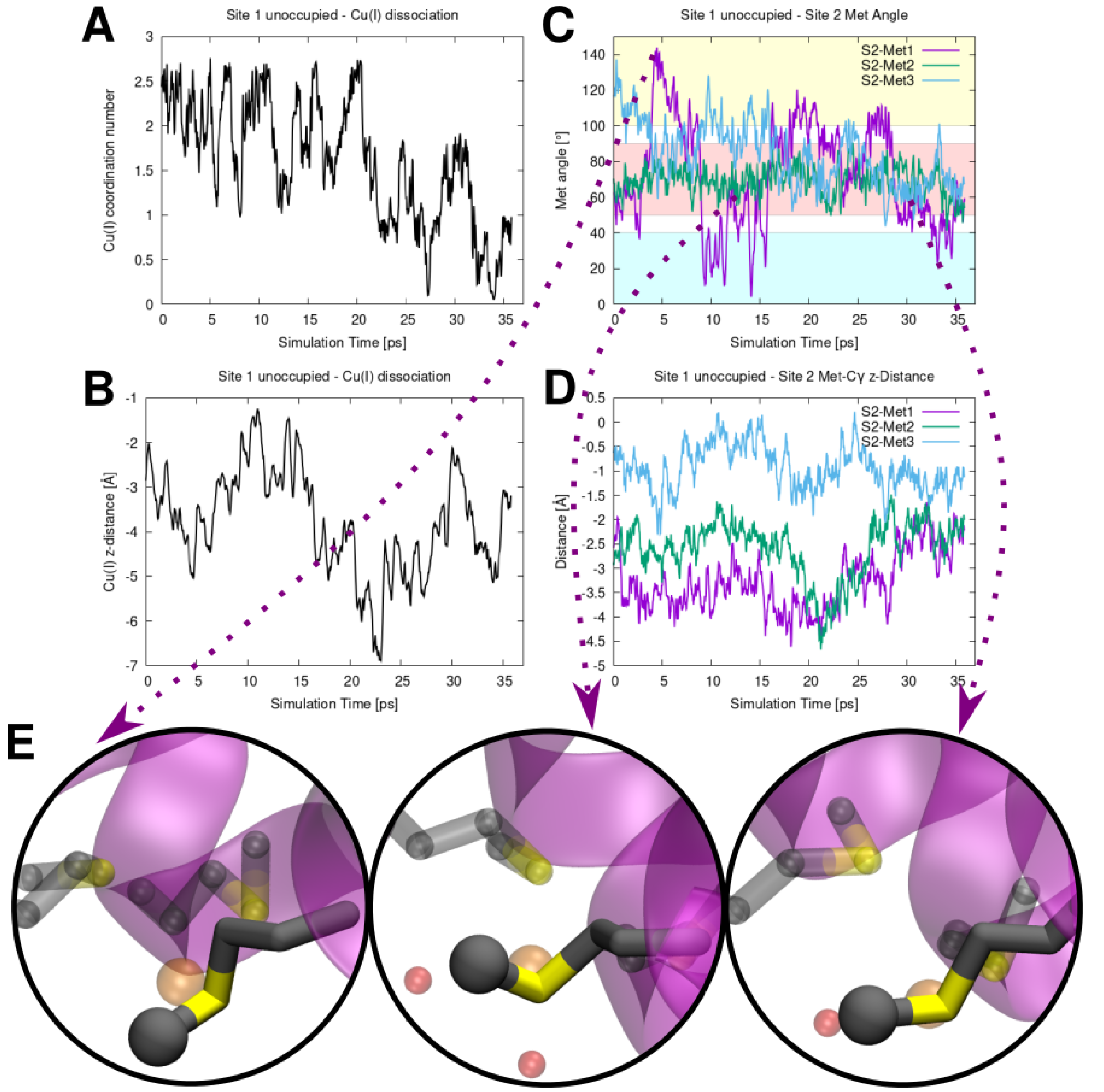

Figure S8. Cu(I) dissociation from Site 2 (S2) in the absence of Cu(I) bound to Site 1. Evolution of the Collective variables (CVs) used in the metadynamics simulation: (A) Coordination number of the Cu(I) ion to the S atoms of the S2-Met triad; (B) projection along the selectivity filter axis (z-axis) of the distance (Å) between the Cu(I) to the center of the filtration layer (defined as the center of of the S2-Met triad Cα atoms). (C) Conformation of the Site 2 Mets measured as the θ angle (°) between the Met Cγ-Cε vector and the vector of the selectivity filtered defined by geometric centers of Site 1 and Site 2

backbone atoms. S2-Met1-3 refer to the methionines of the bottom (intracellular-exposed Met-triad). Areas of the plot, corresponding to the inward, in-plane and outward conformations are highlighted in cyan, red and yellow, respectively. (D) Z-projection of distance (Å) of the Site 2 Met@Cγ to the center of the selectivity filter defined as the center of Site 1 and Site 2 Met@Cα atoms. (E) Close-ups of the states corresponding to the conformational change of the Site 2-Met1.

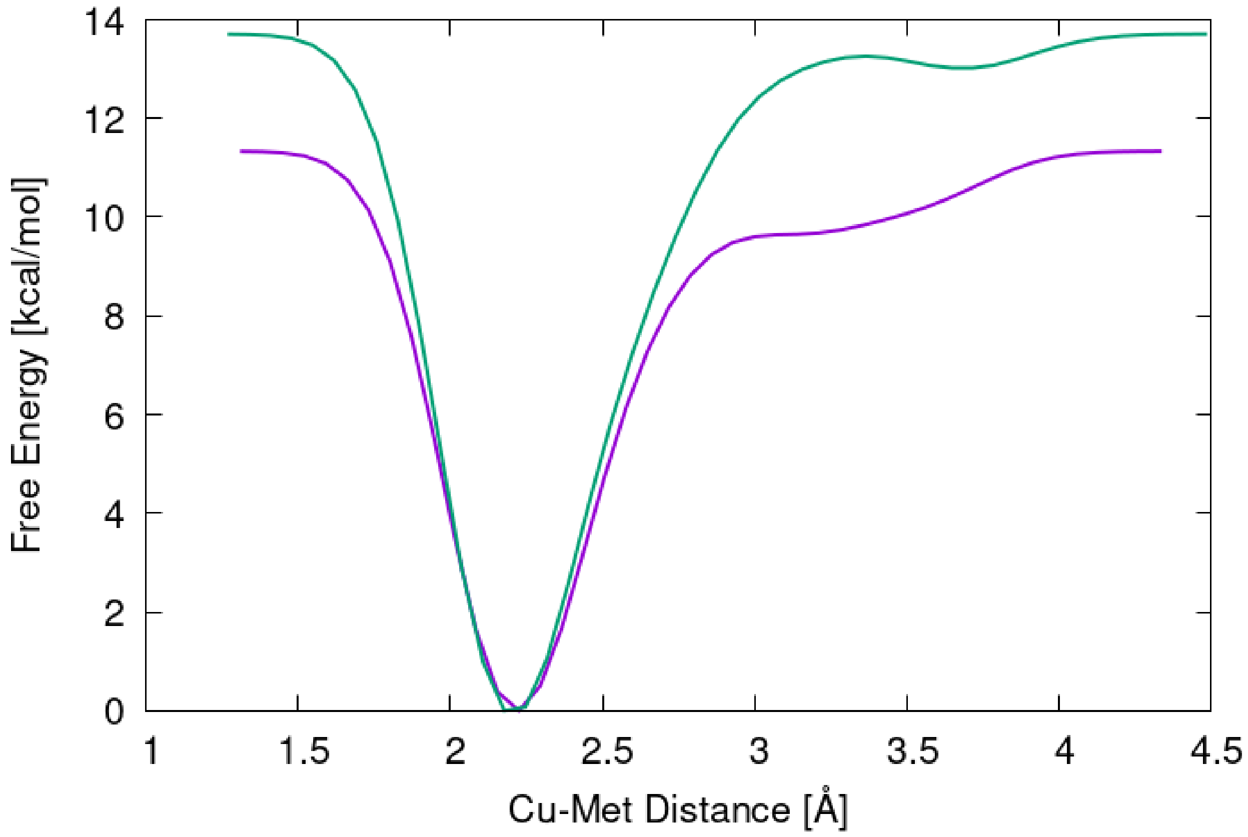

Figure S9. Free energy profile (kcal/mol) of the Cu(I) dissociation from Site 2 in absence of Cu(I) in Site 1 as a function of Cu(I) distance (Å) to the last coordinating Met@S atoms from the two repeats of the simulation (green and purple line).

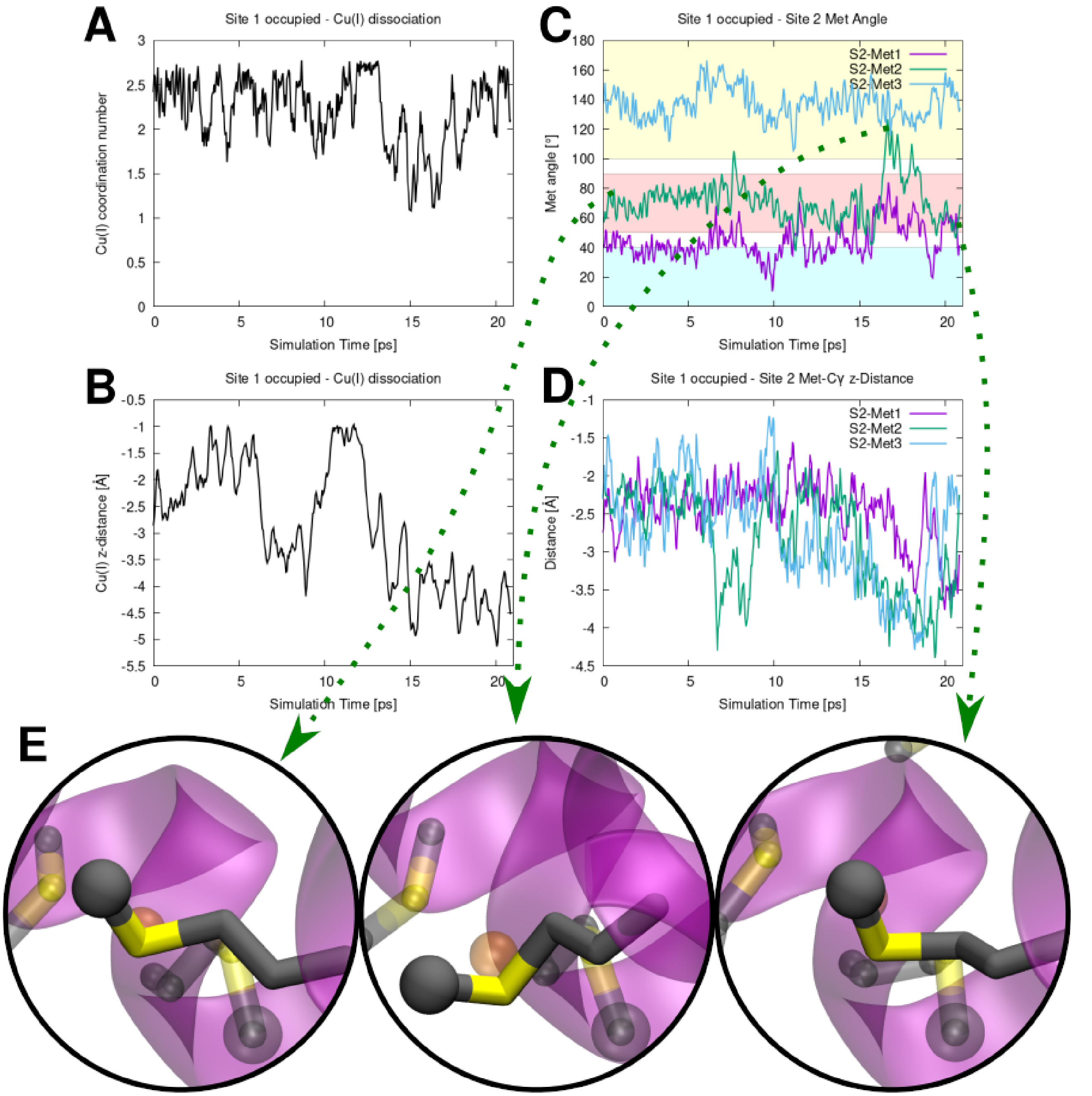

Figure S10. Cu(I) dissociation from Site 2 with a Cu(I) ion bound to Site 1. Evolution of the Collective variables (CVs) used in the metadynamics simulation: (A) Coordination number of the Cu(I) ions to the S atoms of the Site 2-Met triad; (B) projection along the selectivity filter axis (z-axis) of the distance (Å) between the Cu(I) to the center of the filtration layer (defined as the center of the Site 2-Met triad Cα atoms). (C) Conformation of the Site 2 Mets measured as the θ angle (°) between the Met Cγ-Cε vector and the vector of the selectivity filtered defined by geometric centers of Site 1 and Site 2 backbone atoms. S2-Met1-3 refer to the methionines of the bottom (intracellular-exposed Met-triad).

Areas of the plot corresponding to the inward, in-plane and outward conformations are highlighted in cyan, red and yellow, respectively. (D) Z-projection of distance (Å) of the Site 2 Met@Cγ to the center of the selectivity filter defined as the center of Site 1 and Site 2 Met@Cα atoms. (E) Close-ups of the states corresponding to the conformational change of Site 2-Met2.

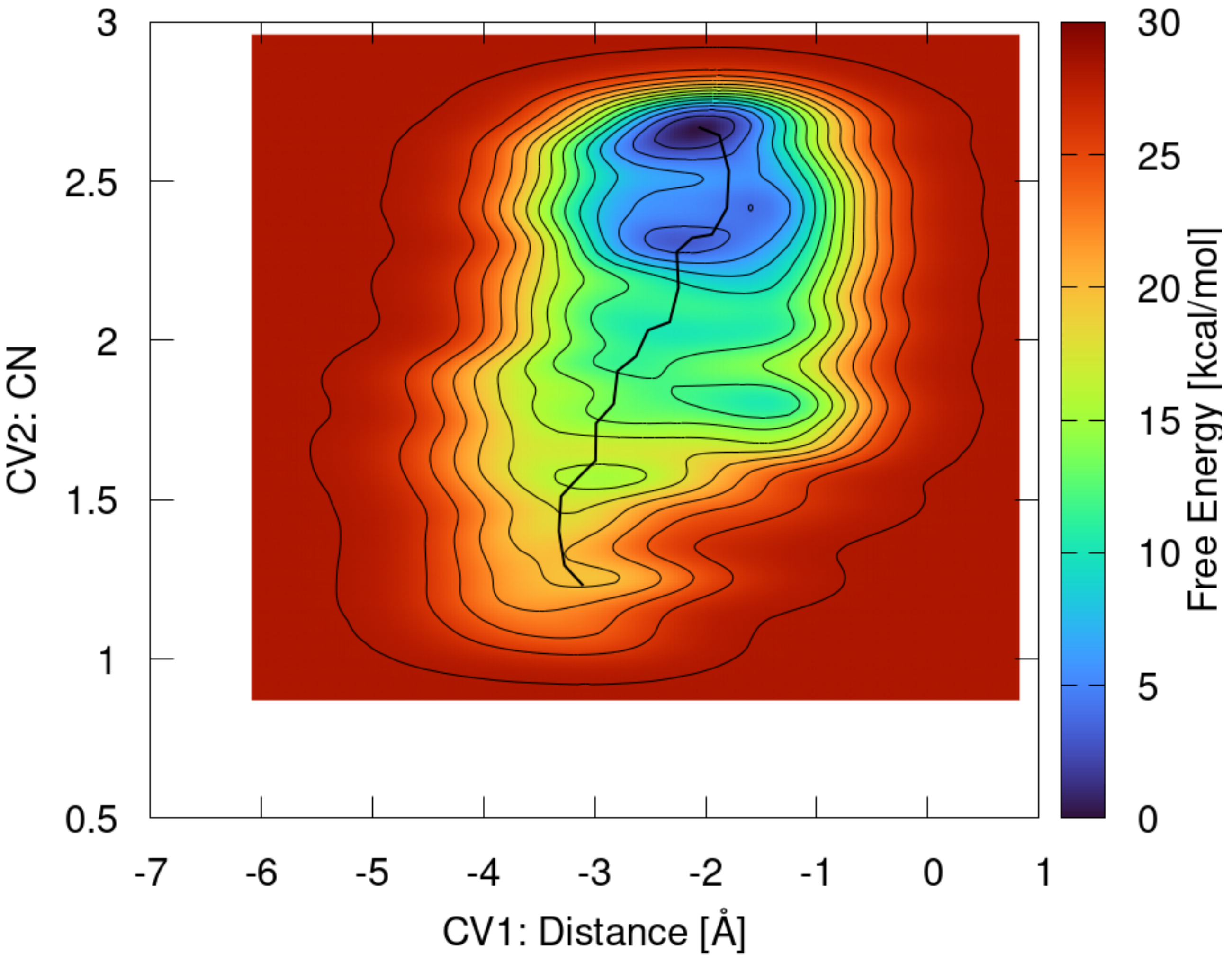

Figure S11. Free energy surface (FES, kcal/mol) of Cu(I) dissociation from Site 2 in the presence of Cu(I) ion bound to Site 1 and with the Site 2 Met-triads restrained to "in-plane" conformation. The FES

is plotted as a function of the two Collective Variable (CVs) used in the metadynamics simulation: CV1 – projection along the z-axis of the distance between the Cu(I) ion and the center of Met-Cα atoms of the Site 2-Met triad; and CV2 – coordination number of Cu(I) with respect to the Site 2-Met triad S atoms. Minimum free energy path is plotted as a black line. The FES is shown from blue to red with isosurface lines drawn every 2.0 kcal/mol.

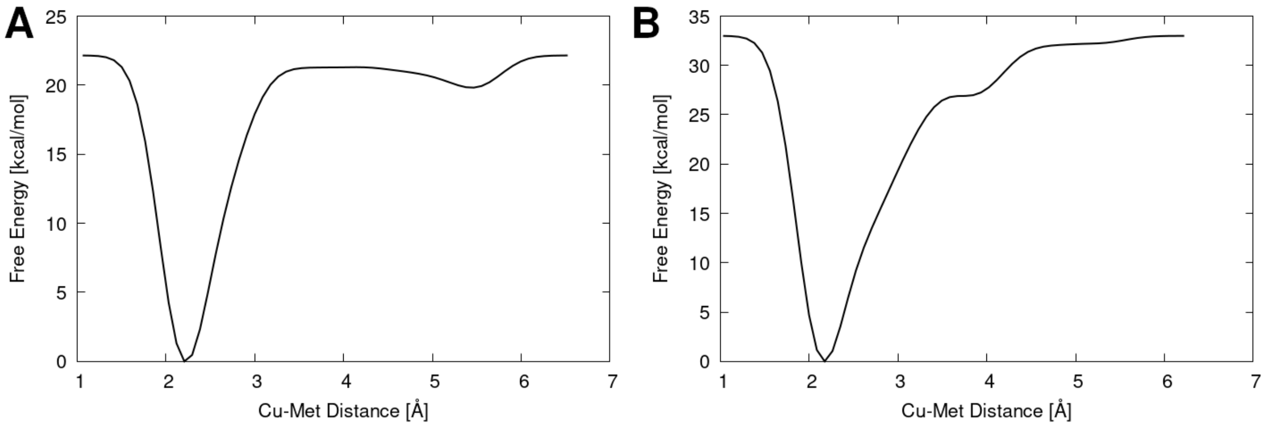

Figure S12. Free energy profiles (FEP, kcal/mol) of the final Cu(I) dissociation step from Site 2 in presence of Cu(I) in Site 1. The FEP is plotted as a function of the distance of the Cu(I) to the last coordinated Met@S atoms of Site 2. (A) All Mets restrained to the IP-conformation. (B) No restraints on the Mets conformation (i.e. one Met is in the O-conformation).

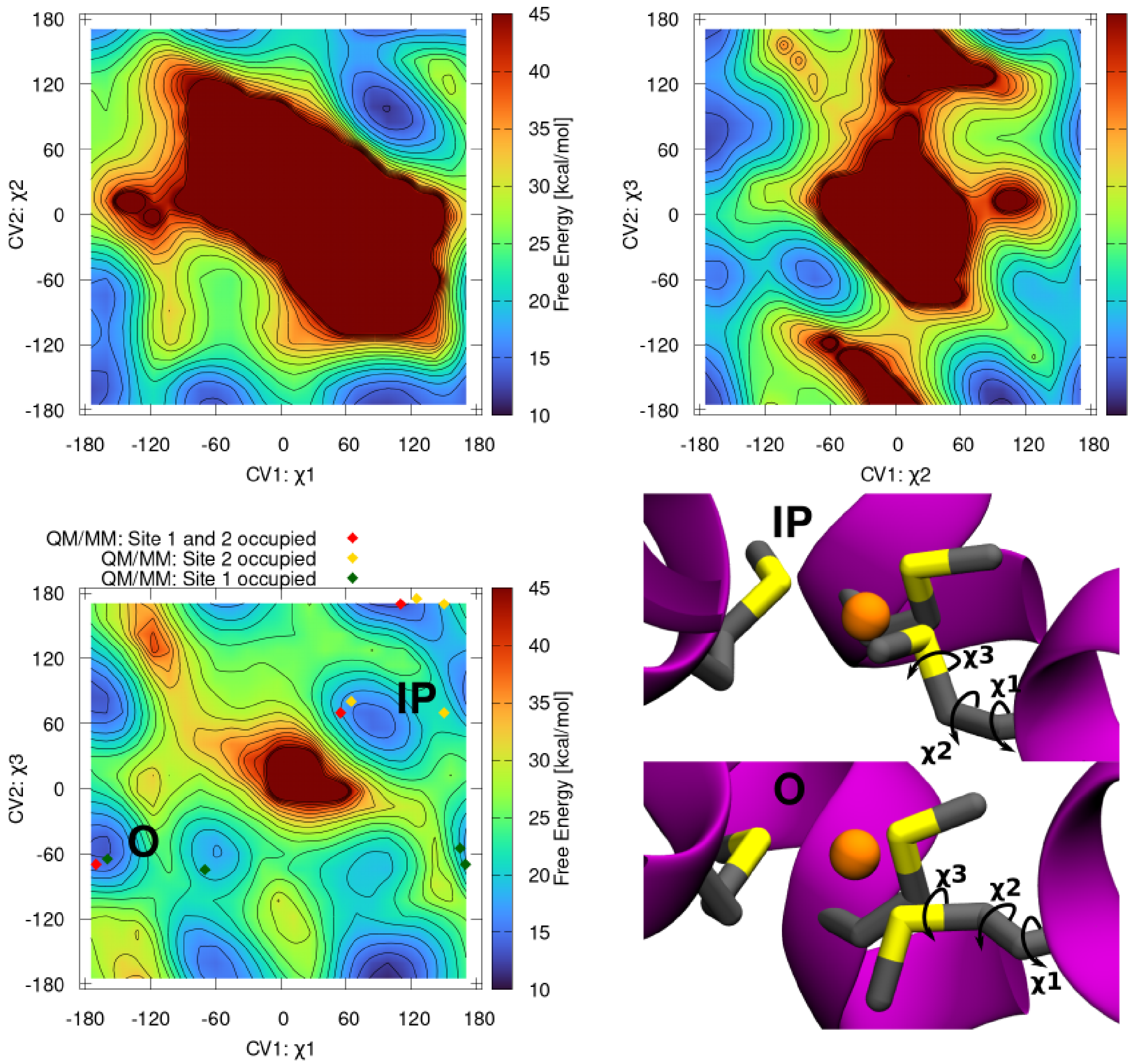

Figure S13. Free energy surfaces (FESs, kcal/mol) of a Site 2 Met conformational landscape as a function of the three torsion angles χ1, χ2 and χ3 as obtained from classical metadynamics simulation in the presence of a Cu(I) ion bound to both Site 1 and Site 2. In this simulation the other S2-Mets are restrained into the IP-conformation. The FESs are shown from blue to red with isosurface lines drawn every 2.0 kcal/mol. The IP- and O-conformations can be described in the FES vs the χ1, χ3 surface, as the flipping involves mainly these two variables. Predominant conformations of the Site 2-Mets observed in QM/MM MD simulation for the CTR1 model in the presence of two Cu(I) ions and the models with single Cu(I) bound to either Site 1 and Site 2 are reported as red, green and yellow dots, respectively.

Movie M1. Translocation of the Cu(I) from Site 1 to Site 2 obtained from the QM/MM metadynamics simulations. CTR1 is shown as magenta α-helices. Met residues are shown in licorice with S and C atoms in yellow and gray, respectively. Hydrogen atoms are omitted for clarity. Cu(I) is depicted as an orange van deer Waals sphere. Cu(I) coordination is shown with orange lines.

Movie M2. Dissociation of the Cu(I) from the Site 2 into the cytosol-exposed CTR1 vestibule from the QM/MM metadynamics simulation with unoccupied Site 1. CTR1 is shown as magenta α-helices. Met residues are shown in licorice with S and C atoms in yellow and gray, respectively. Water molecules are shown as red spheres. Hydrogen atoms are omitted for clarity. Cu(I) is depicted as an orange van deer Waals sphere. Cu(I) coordination is shown with orange lines.

References

1. F. Ren, *et al.*, X-ray structures of the high-affinity copper transporter Ctr1. *Nat. Commun.* **10**, 1386 (2019).

2. B. Webb, A. Sali, Comparative protein structure modeling using MODELLER. *Curr. Protoc. Bioinforma.* **54**, 5–6 (2016).

3. S. Jo, T. Kim, V. G. Iyer, W. Im, CHARMM-GUI: a web-based graphical user interface for CHARMM. *J. Comput. Chem.* **29**, 1859–1865 (2008).

4. E. L. Wu, *et al.*, CHARMM-GUI membrane builder toward realistic biological membrane simulations. *J. Comput. Chem.* **35**, 1997–2004 (2014).

5. M. A. Lomize, I. D. Pogozheva, H. Joo, H. I. Mosberg, A. L. Lomize, OPM database and PPM web server: resources for positioning of proteins in membranes. *Nucleic Acids Res.* **40**, D370–D376 (2012).

6. J. A. Maier, *et al.*, ff14SB: Improving the Accuracy of Protein Side Chain and Backbone Parameters from ff99SB. *J. Chem. Theory Comput.* **11**, 3696–3713 (2015).

7. C. J. Dickson, *et al.*, Lipid14: The Amber Lipid Force Field. *J. Chem. Theory Comput.* **10**, 865–879 (2014).

8. W. L. Jorgensen, J. Chandrasekhar, J. D. Madura, R. W. Impey, M. L. Klein, Comparison of simple potential functions for simulating liquid water. *J. Chem. Phys.* **79**, 926 (1983).

9. I. S. Joung, T. E. Cheatham, Determination of Alkali and Halide Monovalent Ion Parameters for Use in Explicitly Solvated Biomolecular Simulations. *J. Phys. Chem. B* **112**, 9020–9041 (2008).

10. B. T. Op't Hol, K. M. Merz, Insights into Cu (I) exchange in HAH1 using quantum mechanical and molecular simulations. *Biochemistry* **46**, 8816–8826 (2007).

11. M. J. Abraham, *et al.*, GROMACS: High performance molecular simulations through multi-level parallelism from laptops to supercomputers. *SoftwareX* **1**, 19–25 (2015).

12. J. Hutter, M. Iannuzzi, F. Schiffmann, J. VandeVondele, CP2K: atomistic simulations of condensed matter systems. *Wiley Interdiscip. Rev. Comput. Mol. Sci.* **4**, 15–25 (2014).

13. G. A. Tribello, M. Bonomi, D. Branduardi, C. Camilloni, G. Bussi, PLUMED 2: New feathers for an old bird. *Comput. Phys. Commun.* **185**, 604–613 (2014).

14. A. D. Becke, Density-functional exchange-energy approximation with correct asymptotic behavior. *Phys. Rev. A* **38**, 3098 (1988).

15. C. Lee, W. Yang, R. G. Parr, Development of the Colle-Salvetti correlation-energy formula into a functional of the electron density. *Phys. Rev. B* **37**, 785 (1988).

16. B. Miehlich, A. Savin, H. Stoll, H. Preuss, Results obtained with the correlation energy density functionals of Becke and Lee, Yang and Parr. *Chem. Phys. Lett.* **157**, 200–206 (1989).

17. Z. Qasem, *et al.*, The pivotal role of MBD4–ATP7B in the human Cu (I) excretion path as revealed by EPR experiments and all-atom simulations. *Metallomics* **11**, 1288–1297 (2019).

18. M. Pavlin, *et al.*, Unraveling the impact of cysteine-to-serine mutations on the structural and functional properties of Cu (I)-binding proteins. *Int. J. Mol. Sci.* **20**, 3462 (2019).

19. J. VandeVondele, J. Hutter, Gaussian basis sets for accurate calculations on molecular systems in gas and condensed phases. *J. Chem. Phys.* **127**, 114105 (2007).

20. S. Goedecker, M. Teter, J. Hutter, Separable dual-space Gaussian pseudopotentials. *Phys. Rev. B* **54**, 1703 (1996).

21. S. Grimme, J. Antony, S. Ehrlich, H. Krieg, A consistent and accurate ab initio parametrization of density functional dispersion correction (DFT-D) for the 94 elements H-Pu. *J. Chem. Phys.* **132**, 154104 (2010).

22. G. Bussi, D. Donadio, M. Parrinello, Canonical sampling through velocity rescaling. *J. Chem. Phys.* **126**, 014101 (2007).

23. P. Janoš, A. Magistrato, All-Atom Simulations Uncover the Molecular Terms of the NKCC1 Transport Mechanism. *J. Chem. Inf. Model.* **61**, 3649–3658 (2021).

24. P. Li, K. M. Merz, MCPB.py: A Python Based Metal Center Parameter Builder. *J. Chem. Inf. Model.* **56**, 599–604 (2016).

25. D. R. Roe, T. E. Cheatham, PTRAJ and CPPTRAJ: Software for Processing and Analysis of Molecular Dynamics Trajectory Data. *J. Chem. Theory Comput.* **9**, 3084–3095 (2013).

26. C. R. Harris, *et al.*, Array programming with NumPy. *Nature* **585**, 357–362 (2020).

27. D. Trapl, V. Spiwok, Analysis of the Results of Metadynamics Simulations by metadynminer and metadynminer3d. *ArXiv Prepr. ArXiv200902241* (2020).
